# Draft Genome Sequence of Polyaromatic Hydrocarbon-Degrading Bacterium *Bacillus subtilis* SR1, Which Has Plant Growth-Promoting Attributes

**DOI:** 10.1128/genomeA.01339-17

**Published:** 2017-12-07

**Authors:** Rhitu Kotoky, L. Paikhomba Singha, Piyush Pandey

**Affiliations:** Department of Microbiology, Assam University, Silchar, India

## Abstract

*Bacillus subtilis* SR1 is a heavy metal-resistant, polyaromatic hydrocarbon-degrading bacterium isolated from rhizospheric soil of contaminated sites. It has the ability to promote plant growth and utilize benzo[*a*]pyrene as a carbon source. This study reports the characteristics of the genome of *B. subtilis* SR1, which contains one circular chromosome (4,093,698 bp).

## GENOME ANNOUNCEMENT

Microbial degradation represents the major route responsible for the ecological recovery of polyaromatic hydrocarbon (PAH)-contaminated sites. Microorganisms can absorb, transform, or degrade PAHs, reduce the mobility and bioavailability of contaminants (1), and break down or mineralize hazardous organic pollutants into less harmful or nontoxic compounds. The bacterial strain *B. subtilis* SR1 was isolated from rhizospheric soil of a petroleum-contaminated site. It was isolated in minimal medium (Bushnell-Haas broth) amended with benzo[*a*]pyrene (BaP) and tested for heavy metal resistance and plant growth-promotion attributes. The bacterium was found to be resistant to different heavy metals and capable of degrading PAHs. Qualitative and quantitative analysis for degradation of BaP was done by high-pressure liquid chromatography (HPLC) and gas chromatography-mass spectroscopy (GC-MS) analysis, as described earlier (2). The strain was examined for plant growth-promotion attributes, such as indole-3-acetic acid production, siderophore production, phosphate solubilization, and hydrogen cyanide production (2).

The draft genome sequence of *B. subtilis* SR1 was obtained by whole-genome shotgun sequencing using one Illumina paired-end library with an average insert size of ~400 bp. The paired-end sequencing libraries were prepared using an Illumina TruSeq nano DNA library prep kit. The DNA was fragmented by Covaris M220, generating a double-stranded DNA fragment with a 3′ or 5′ overhang. The fragments were then subjected to end repair, followed by adapter ligation to the fragments. The products were then PCR amplified with the index primer, as described in the kit protocol, and sequenced using the NextSeq 500 platform. The NextSeq 500 paired-end sequencing run generated ~1 Gb of raw reads.

The sequenced raw data were processed to obtain high-quality clean reads using Trimmomatic version 0.35 to remove adapter sequences, ambiguous reads, and low-quality sequences. These reads were trimmed using a quality score threshold of 20 and a length cutoff of 20 bp. Reference-guided assembly of the sample was performed using SAMtools (3). The procedure for genome annotation was done with the Rapid Annotations using Subsystems Technology (RAST) server and the NCBI Prokaryotic Genome Annotation Pipeline (PGAP) (https://www.ncbi.nlm.nih.gov/genome/annotation_prok) (4).

The genome of *Bacillus subtilis* SR1 consists of one circular chromosome (4,093,698 bp) with a GC content of 44.01%, as well as 4,098 protein-coding genes; 83 tRNAs; 120 RNAs, including 22 rRNAs; and 106 pseudogenes.

### Accession number(s).

The draft whole-genome shotgun sequence of *B. subtilis* SR1 has been deposited in GenBank under the accession number CP021985 and BioProject number PRJNA388844.
